# Dietary Utilization Drives the Differentiation of Gut Bacterial Communities between Specialist and Generalist Drosophilid Flies

**DOI:** 10.1128/spectrum.01418-22

**Published:** 2022-07-11

**Authors:** Jia-Syuan Chen, Shun-Chern Tsaur, Chau-Ti Ting, Shu Fang

**Affiliations:** a Department of Life Science, National Taiwan University, Taipei, Taiwan, Republic of China; b Center for General Education, National Taiwan University, Taipei, Taiwan, Republic of China; c Institute of Ecology and Evolutionary Biology, National Taiwan University, Taipei, Taiwan, Republic of China; d Center for Biotechnology, National Taiwan University, Taipei, Taiwan, Republic of China; e Center for Developmental Biology and Regenerative Medicine, National Taiwan University, Taipei, Taiwan, Republic of China; f Genome and Systems Biology Degree Program, National Taiwan University and Academia Sinica, Taipei, Taiwan, Republic of China; g Biodiversity Research Center, Academia Sinica, Taipei, Taiwan, Republic of China; Brigham Young University

**Keywords:** *Bacteroidetes*, *Colocasiomyia*, complex carbohydrate utilization, *Drosophila*, gut bacteria, *Firmicutes*, *Proteobacteria*, specialists

## Abstract

Gut bacteria play vital roles in the dietary detoxification, digestion, and nutrient supplementation of hosts during dietary specialization. The roles of gut bacteria in the host can be unveiled by comparing communities of specialist and generalist bacterial species. However, these species usually have a long evolutionary history, making it difficult to determine whether bacterial community differentiation is due to host dietary adaptation or phylogenetic divergence. In this regard, we investigated the bacterial communities from two Araceae-feeding *Colocasiomyia* species and further performed a meta-analysis by incorporating the published data from *Drosophila* bacterial community studies. The compositional and functional differentiation of bacterial communities was uncovered by comparing three (Araceae-feeding, mycophagous, and cactophilic) specialists with generalist flies. The compositional differentiation showed that *Bacteroidetes* and *Firmicutes* inhabited specialists, while more *Proteobacteria* lived in generalists. The functional prediction based on the bacterial community compositions suggested that amino acid metabolism and energy metabolism are overrepresented pathways in specialists and generalists, respectively. The differences were mainly associated with the higher utilization of structural complex carbohydrates, protein utilization, vitamin B_12_ acquisition, and demand for detoxification in specialists than in generalists. The complementary roles of bacteria reveal a connection between gut bacterial communities and fly dietary specialization.

**IMPORTANCE** Gut bacteria may play roles in the dietary utilization of hosts, especially in specialist animals, during long-term host-microbe interaction. By comparing the gut bacterial communities between specialist and generalist drosophilid flies, we found that specialists harbor more bacteria linked to complex carbohydrate degradation, amino acid metabolism, vitamin B_12_ formation, and detoxification than do generalists. This study reveals the roles of gut bacteria in drosophilid species in dietary utilization.

## INTRODUCTION

Dietary specialization has been reported in many lineages of animals (1). Recent studies also demonstrated that gut bacteria play an indispensable role in dietary detoxification, complex carbohydrate digestion, and nutritional supplementation of their animal hosts (2). In dietary detoxification, genes involved in the degradation process of plant secondary metabolites have been identified to be enriched in the gut microbiomes of specialist animals, such as pandas (3), koalas (4), and several insect orders (5). In complex carbohydrate digestion, genes involved in the metabolism of complex carbohydrates are enriched in the gut microbiomes of specialists, such as baleen whales, which feed on copepods (6), bees, which feed on pollen (7), and termites, which feed on wood (8). In nutritional supplementation, data show that some endosymbiotic microbes provide essential amino acids and vitamins to insects, such as aphids (9) and tsetse flies (10). These functional complements of gut bacteria may facilitate host-symbiont interactions and coevolution in the dietary specialization of animal hosts (11).

The strong bond between drosophilids and microbes makes the former an ideal model to study the host-microbe interaction. Microbes can decompose macromolecules of the decaying material consumed by flies. During the rotting process, microbes can also generate volatiles to attract flies (12). *Drosophila*-associated microbes may rely on adult flies to disperse. Thus, *Drosophila* often carries and transfers the microbes to new substrates through the fecal-oral route (13, 14). Drosophilids can be categorized as either generalists or specialists, depending on dietary preference. Generalists feed and breed on a wide variety of rotting plant tissues, whereas specialists adapt to either a specific part of plants, such as flowers and tree saps, or a specific group of hosts (15). Among the specialists, the cactophilic and mycophagous *Drosophila* groups are the most well-studied groups in terms of dietary adaptation. The food sources of specialist flies usually contain toxic secondary metabolites, such as a high concentration of alkaloids in cacti (16) and α-amanitin in some mushrooms (17, 18). In addition to the adaptation of toxin tolerance, the nutritional adaptability also varies between specialist and generalist flies (19, 20). The generalists utilize diets ranging from low to high sugar content, whereas the specialists consume diets with low sugar and high protein (21). These dietary differences may drive the differentiation of the gut bacterial community between generalist and specialist flies.

**FIG 1:**
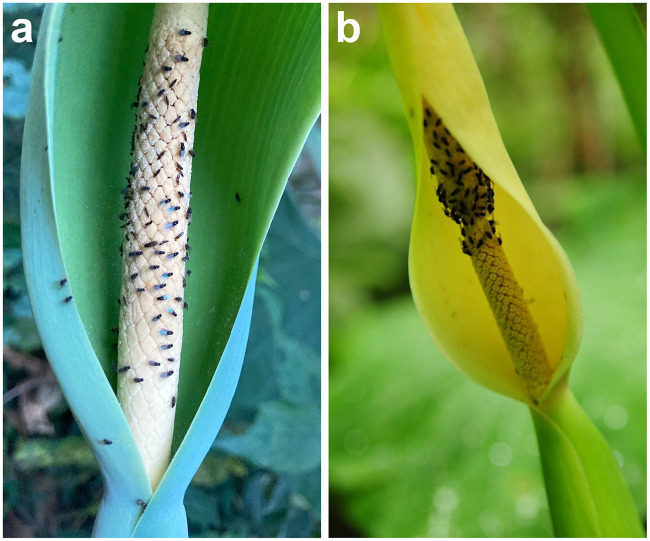
*Colocasiomyia* flies on the flowers of host plants (a) *Alocasia odora* and (b) *Colocasia formosana* (photo courtesy of Kuo-Fang Chung).

A wide range of topics on the gut bacterial communities in *Drosophila* species have been covered in previous studies. These studies have shown that dietary, environmental, phylogenetic, and stochastic effects can influence the assembling of gut bacterial communities (22–29). Among these effects, several studies in natural populations consistently suggest that diet is more influential than other factors. For example, different compositions of bacterial communities have been observed in fruit and flower feeders (22). The bacterial communities of Drosophila melanogaster and Drosophila simulans on the same food resources were more similar than those between the same fly species on different foods (27). As specialists have adapted to diets with higher toxins, higher proteins, and lower sugars than generalists (19–21), specialists are likely to share similar compositions of bacterial communities. To test this idea, we investigated the differences between gut microbial communities of generalists and specialists by combining the available *Drosophila* data and newly studied community data of two *Colocasiomyia* species. Like most *Colocasiomyia* species specializing in 1 or 2 Araceae species (30), both Colocasiomyia alocasiae and Colocasiomyia xenalocasiae complete their life cycles exclusively on their hosts (Fig. 1). The adult flies feed, mate, and oviposit on the inflorescences. The larvae develop and pupate in the enclosed pistillate part of the inflorescences and emerge during the fruiting time of the plants. The unique feeding biology of *Colocasiomyia* enables us to study the relationship between dietary specialization and gut bacterial communities.

In this study, we first surveyed the gut microbial communities of two *Colocasiomyia* species collected from natural populations to evaluate the influences of fly diet, species, and sex on bacterial compositions for four consecutive years. We then performed a meta-analysis of bacterial communities of generalist and specialist flies to ask whether the similarity of bacterial communities matched more with the dietary utilization or with the fly phylogeny. Finally, the bacterial communities between specialists and generalists were compared for their compositional and functional differences to infer the possible roles involved in the dietary specialization of drosophilid flies.

## RESULTS

### Bacterial communities in *Colocasiomyia* flies and host plants.

The gut bacterial communities of specialist *Colocasiomyia* flies were revealed by analyzing the V3-V4 variable regions of 16S rRNA genes from wild-caught adults of *C. alocasiae* and *C. xenalocasiae* and two host plants, namely, Alocasia odora and Colocasia formosana. A total of 2,277,187 raw reads were filtered to 726,387 and 111,579 high-quality reads in flies and host plants, respectively (Fig. S1). These reads were assigned to operational taxonomic units (OTUs) based on a similarity threshold of 97%. Two community samples from the host plants were discarded because of insufficient reads. OTUs with relative abundances less than 0.02% were removed, yielding a total of 123 OTUs (40 ± 2 OTUs per sample) in flies and 117 OTUs (45 ± 5 OTUs per sample) in host plants (see Table S1 in the supplemental material). Approximately half (50.1 ± 8.9%) (Table S2) of fly bacterial OTUs were specific to flies and undetected in plant samples. The OTU abundance between paired samples of flies and host plants was either negatively or not correlated (Spearman’s rank correlation) (Fig. S2). Nonmetric multidimensional scaling (NMDS) based on Bray-Curtis dissimilarity and principal-coordinate analysis (PCoA) based on UniFrac analysis showed that the first coordinate (NMDS 1 and PCoA 1) separated the bacterial communities between flies and host plants (Mann-Whitney *U* test, *P < *0.05) (Fig. S3). These results suggested that the bacterial communities of flies and plants influenced each other but maintained different compositions. The differences in bacterial communities between flies and their host plants might be contributed to by microenvironments within fly guts and around plants.

### A high proportion of unclassified bacteria were detected in the bacterial communities of *Colocasiomyia* flies.

**FIG 2:**
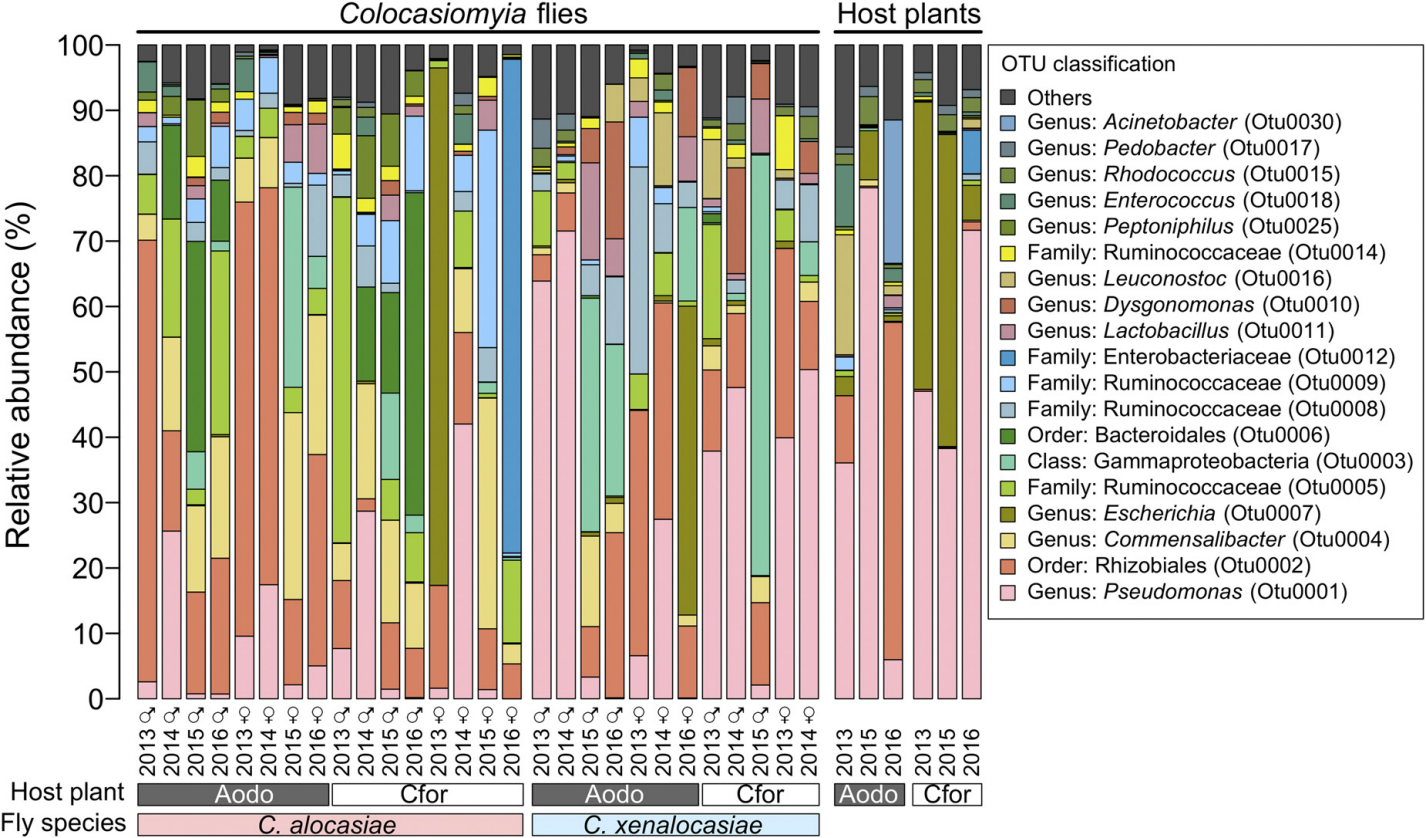
Relative abundances of bacteria identified in *Colocasiomyia* fly guts and on host plants of flies. Aodo, *Alocasia odora*; Cfor, *Colocasia formosana*.

The bacterial communities of *Colocasiomyia* flies and host plants were composed of 19 major OTUs (relative abundance greater than 1% in a sample) (Fig. 2). Individual flies and plants harbored 10 ± 0 and 8 ± 1 major OTUs (Table S1), respectively. Notably, a high proportion of major OTUs (42.1%, 8/19) were unclassified; i.e., OTUs could not be properly assigned to any known genus. Among the eight unclassified OTUs, the most abundant OTU (Otu0002, 20.2% ± 18.2% of the bacterial communities) belongs to *Rhizobiales*, four could be assigned to *Ruminococcaceae* (Otu0005, 7.5% ± 11.1%; Otu0008, 4.0% ± 6.7%; Otu0009, 4.3% ± 6.0%; Otu0014, 1.5% ± 1.7%; total, 17.3% ± 14.3%), and the other three were assigned to *Gammaproteobacteria* (Otu0003, 7.3% ± 14.7%), *Bacteroidales* (Otu0006, 4.9% ± 11.4%), and *Enterobacteriaceae* (Otu0012, 2.7% ± 14.1%).

### Multiple factors contributed to the variation in gut bacterial communities in *Colocasiomyia* flies.

**FIG 3:**
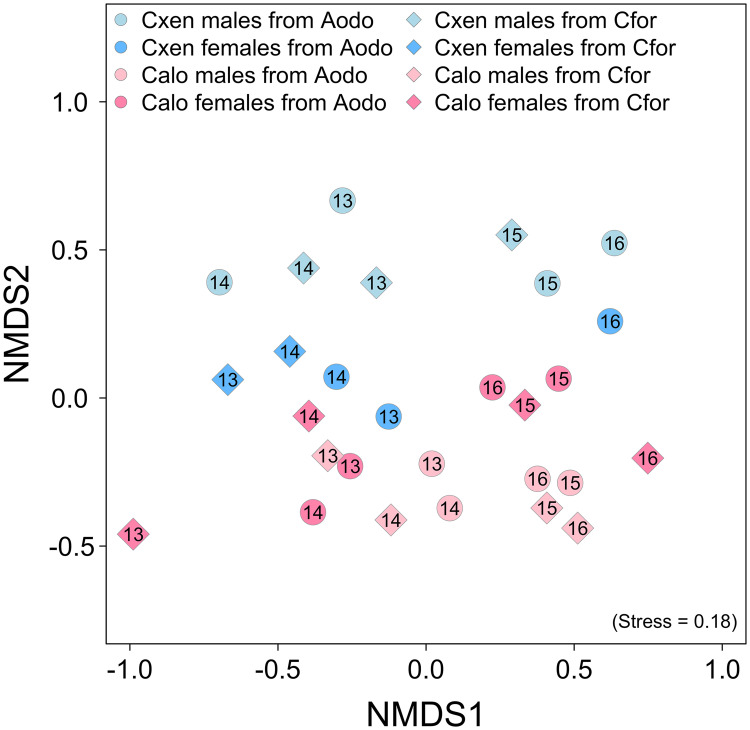
NMDS plot of gut bacterial communities in *Colocasiomyia* flies. The numbers 13 through 16 indicate samples collected in 2013 through 2016. Calo, *Colocasiomyia alocasiae*; Cxen, *C. xenalocasiae*; Aodo, *Alocasia odora*; Cfor, *Colocasia formosana*.

To evaluate the effects of different factors contributing to the variation in bacterial communities in *Colocasiomyia* flies, the beta diversity of the samples in different years, fly species, host plants, and fly sex was first quantified by the Bray-Curtis dissimilarity, Jaccard distance, and Theta YC distance with or without OTU presence and then analyzed by permutational multivariate analysis of variance (Adonis function). The four variables each contributed 9.4 to 23.6%, 2.2 to 13.2%, 3.0 to 6.2%, and 1.9 to 4.8% of the total variation in fly bacterial communities (Table 1). Similarly, nonmetric multidimensional scaling (NMDS) plots based on the Bray-Curtis dissimilarity of the communities showed a clean separation of bacterial communities between years and fly species (Fig. 3); NMDS 1 divided the 2013/2014 and 2015/2016 samples, and NMDS 2 separated the two fly species samples. Although the major bacterial taxa at all taxonomic levels were similar in the two fly species, all these dominant bacteria fluctuated over time (Fig. S4). For example, the relative abundance of OTU0001 (Pseudomonas) within the 4 years were 5.3%, 28.6%, 1.6%, and 1.5% in *C. alocasiae* and 37.3%, 48.9%, 2.9%, and 0.2% in *C. xenalocasiae*. These patterns were repeatedly observed for all predominant taxa. The results suggested that the variation in bacterial communities in these two closely related *Colocasiomyia* species with similar host plants was mainly affected by environmental fluctuations over the years.

**TABLE 1:** Comparison of bacterial community composition with permutational multivariate analysis of variance (Adonis)

Factor	Weighted UniFrac		Unweighted UniFrac		Bray-Curtis dissimilarity		Jaccard distance		Theta YC distance	
	*R* ^2^	*P*	*R* ^2^	*P*	*R* ^2^	*P*	*R* ^2^	*P*	*R* ^2^	*P*
Yr	0.094	0.706	0.107	0.871	0.191	0.010	0.236	0.001	0.199	0.155
Fly species	0.022	0.819	0.039	0.189	0.132	0.002	0.099	0.002	0.118	0.101
Host plant	0.062	0.087	0.046	0.005	0.039	0.361	0.030	0.685	0.055	0.047
Fly sex	0.019	0.895	0.034	0.975	0.048	0.232	0.020	0.955	0.048	0.047

### The diversity of bacterial communities in drosophilids was associated with diet.

**FIG 4:**
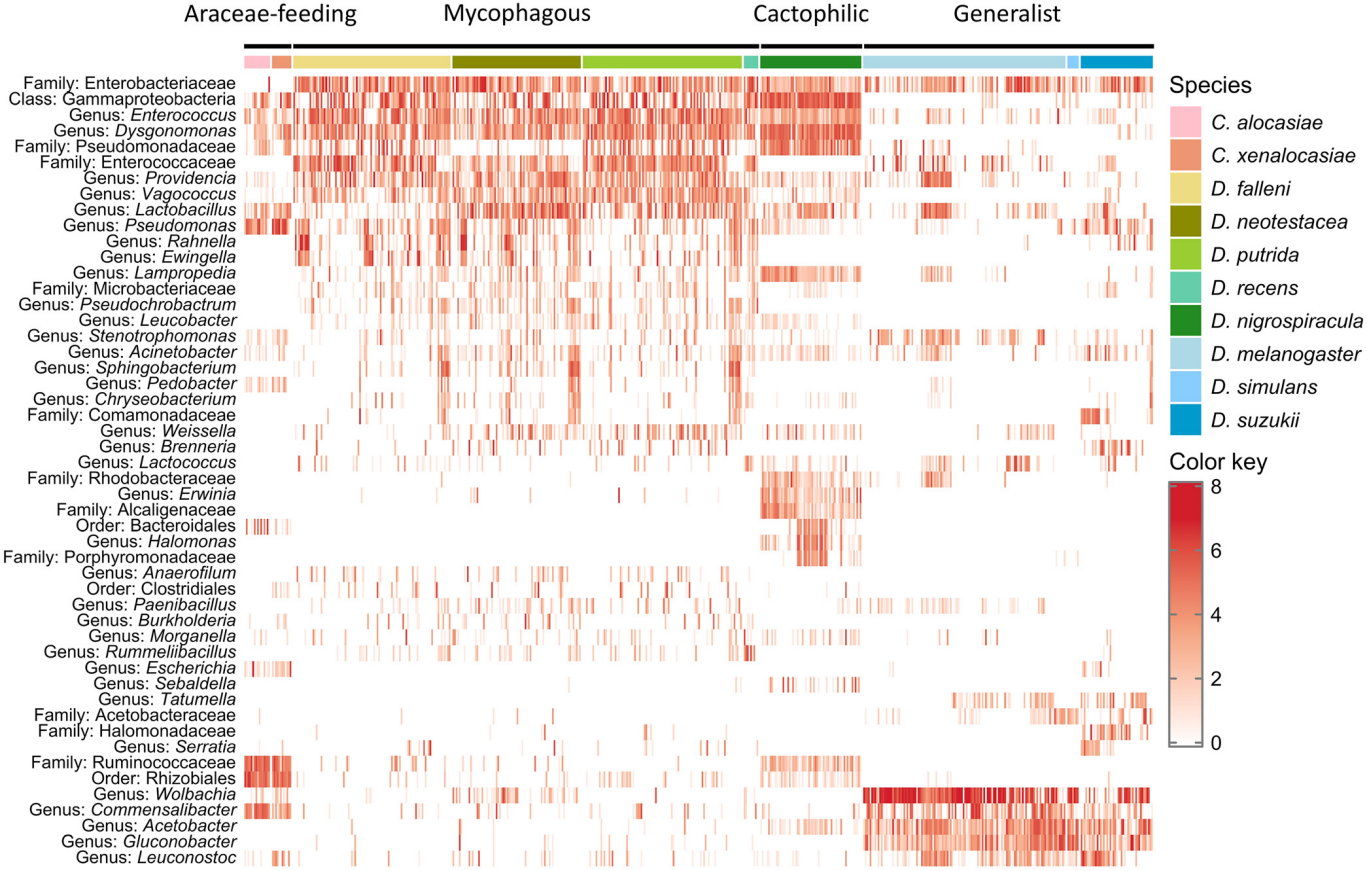
Heat map of the 50 most abundant bacterial genera in specialist and generalist drosophilid species. Generalists include Drosophila melanogaster, *D. simulans*, and *D. suzukii*. Specialists include Araceae-feeding *Colocasiomyia alocasiae* and *C. xenalocasiae*; mycophagous *D. falleni*, *D. neotestacea*, *D. putrida*, and *D. recens*; and cactophilic *D. nigrospiracula*.

**FIG 5:**
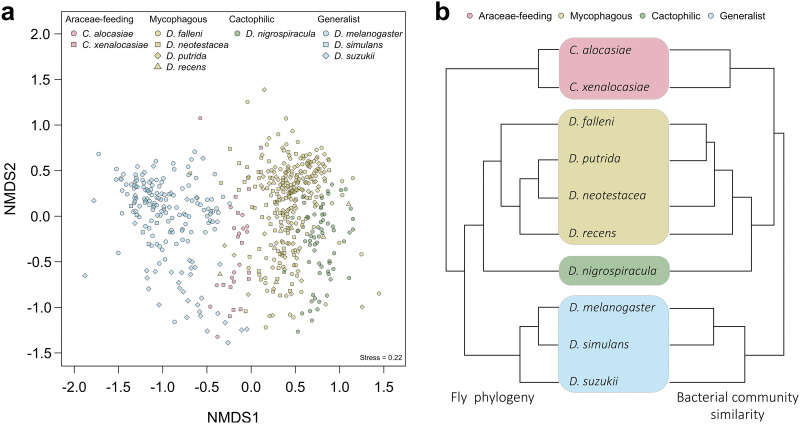
Similarity of gut bacterial communities and fly phylogeny in generalist and specialist drosophilid species. (a) NMDS plot of gut bacterial communities using Bray-Curtis distances. (b) Comparison between the fly phylogeny (95, 96) and the bacterial community dendrogram reconstructed by UPGMA clustering based on the Bray-Curtis distances of bacterial communities between fly species.

To elucidate dietary effects on the bacterial communities of different fly species, we compared the bacterial communities of specialists and their closely related generalists by NMDS analysis based on Bray-Curtis dissimilarity. Seven specialist species, including two Araceae-feeding species (*C. alocasiae* and *C. xenalocasiae*), four mycophagous species (Drosophila falleni, D. neotestacea, D. putrida, and D. recens), one cactophilic species (D. nigrospiracula), and three generalist species (D. melanogaster, D. simulans, and D. suzukii) were analyzed. The results showed that the bacterial communities could be sorted into four groups, consistent with dietary types. A clear separation of bacterial communities between generalist and specialist species was also detected along NMDS 1 (Fig. 4 and 5a). The communities of fly species had a staggered distribution within each dietary group instead of being clustered by fly species (Fig. 5a). Given the different sample sizes among species, the NMDS analysis was performed using both a resampling approach with the smallest sample size and the average bacterial community of each species (see Materials and Methods). The clean separation between generalists and specialists was consistently shown with both approaches (Fig. S5 and S6). The average communities were also used to test if the bacterial community clusters are concordant with species phylogeny. The UPGMA (unweighted pair group method using average linkages) results show that the similarity of the bacterial communities did not fully match the fly phylogeny (Fig. 5b). The gut bacterial communities of Araceae-feeding *Colocasiomyia* species were clustered with those of mycophagous and cactophilic *Drosophila* species, whereas the gut bacterial communities of the generalist *Drosophila* were grouped into another branch. In addition, within the mycophagous species, the gut bacterial community of the relatively distant *D. falleni* was clustered with that of *D. putrida* but not with that of *D. recens* (Fig. 5b). The results suggest that the fly bacterial communities are more influenced by dietary preference than phylogenetic relationships.

**FIG 6:**
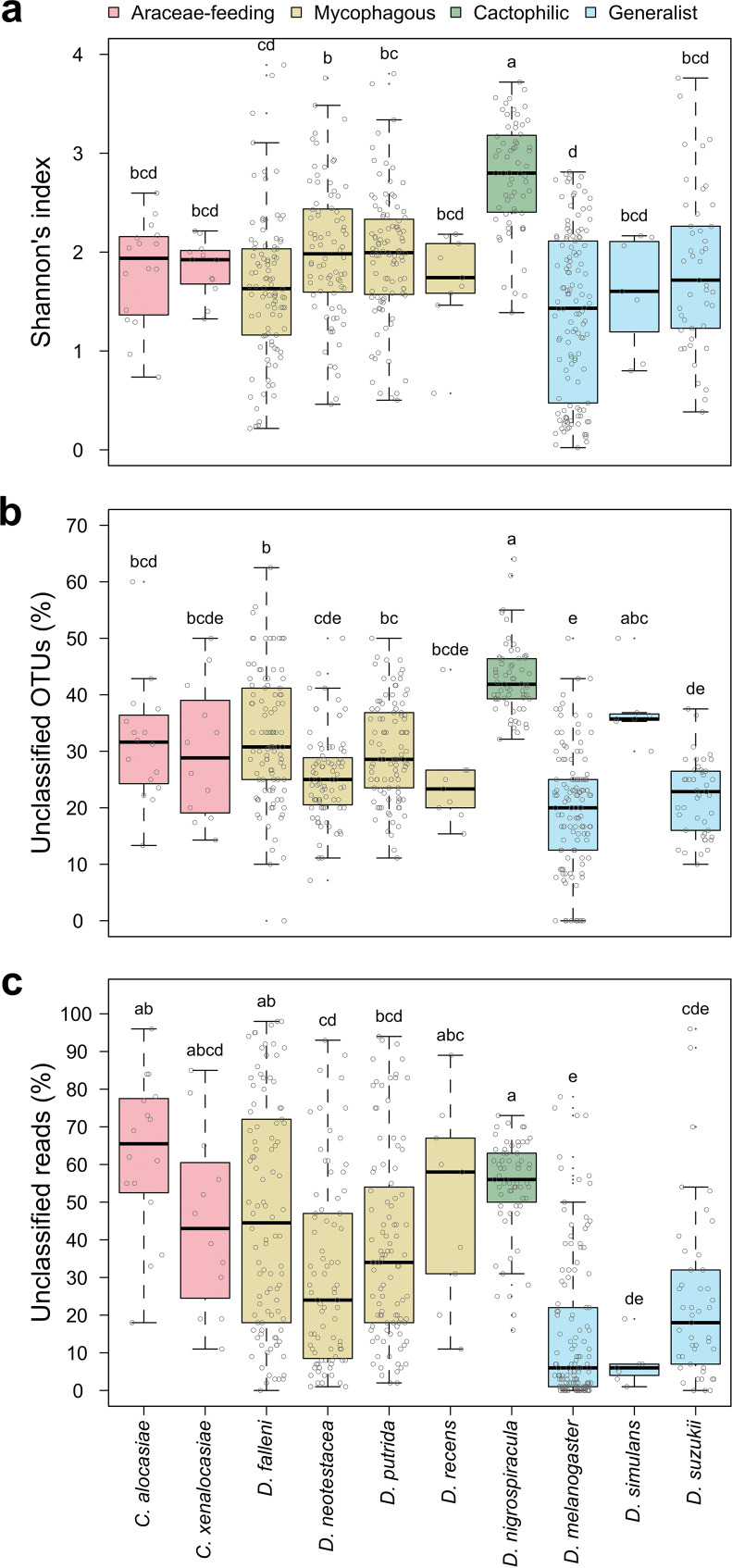
Shannon’s index and unclassified reads of gut bacterial communities in specialist and generalist drosophilid species. (a) Shannon’s index. (b) Percent unclassified OTUs. (c) Percent unclassified reads. Different letters above the boxes indicate significant differences at a *P* value of <0.05 determined by the Kruskal-Wallis test followed by Dunn’s test with the Bonferroni correction.

Gut bacterial diversity based on OTU abundance was quantified by Shannon’s index. Specialists tended to have higher diversity than did generalists (Fig. 6a). As a large number of bacterial communities were sampled from multiple species, we observed a wide range of community diversities in both specialists and generalists. Among the specialists, the cactophilic *Drosophila* exhibited the highest community diversities, while the mycophagous *Drosophila* and the Araceae-feeding *Colocasiomyia* showed intermediate community diversity. In contrast, the three generalists had an intermediate to low community diversity. The differences in community diversity between specialists and generalists were more significant when unclassified or novel bacterial OTUs and reads were compared. In general, specialists had a higher proportion of unclassified bacterial OTUs than did generalists: 35.0% ± 8.8%, 27.9% ± 7.5%, 47.1% ± 3.2%, and 25.1% ± 9.5% in *Colocasiomyia*, mycophagous, cactophilic, and generalist flies, respectively (Fig. 6b). Similarly, specialists had a higher abundance of reads from the unclassified bacteria than did generalists: 54.9% ± 23.5%, 39.2% ± 27.7%, 54.2% ± 12.9%, and 17.0% ± 20.4% in *Colocasiomyia*, mycophagous, cactophilic, and generalist flies, respectively (Fig. 6c). The different abundance of novel bacteria was not biased by the different investigation levels among fly species, because similar to the well-studied generalist D. melanogaster, the two less-studied generalist species also had a lower abundance of novel reads.

### Compositional differentiation of bacterial communities between specialists and generalists.

**FIG 7:**
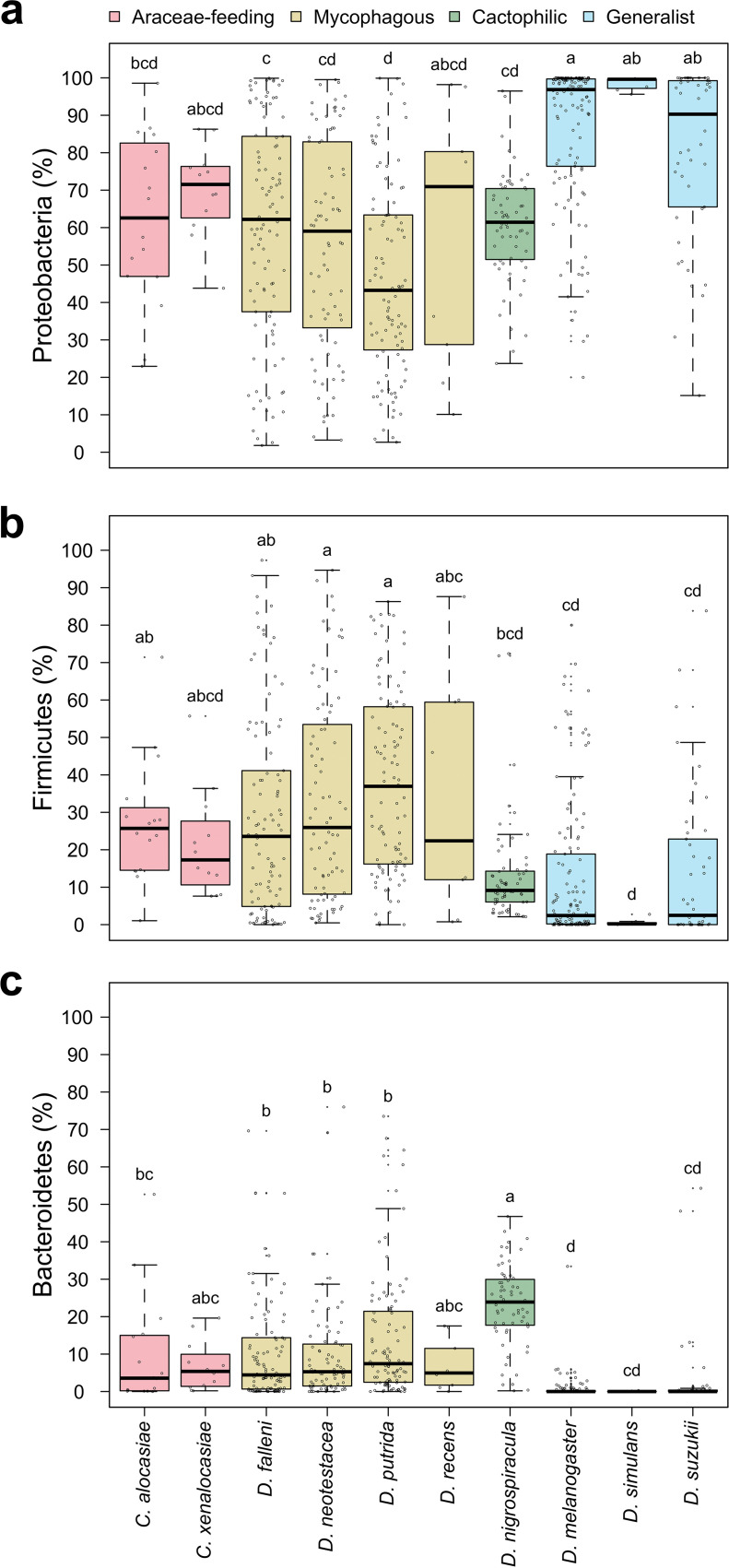
Relative abundances of bacterial phyla appeared in gut bacterial communities of specialist and generalist drosophilid species. Gut bacterial communities of drosophilids are composed mainly of (a) *Proteobacteria*, (b) *Firmicutes*, and (c) *Bacteroidetes*. Different letters above the boxes indicate significant differences at a *P* value of <0.05 determined by the Kruskal-Wallis test followed by Dunn’s test with the Bonferroni correction.

As the bacterial communities of generalists and specialists formed two distinct groups, we asked which bacteria contributed the most to this differentiation. The bacterial community of the drosophilids was composed mainly of *Proteobacteria* (67.6% ± 23.8%), *Firmicutes* (22.2% ± 22.4%), and *Bacteroidetes* (8.5% ± 11.9%). The relative abundances of the three predominant phyla were significantly different between generalist and specialist flies. Generalists harbored more *Proteobacteria* than specialists (85.3% ± 20.3% versus 55.7% ± 26.0%), whereas specialists had more *Firmicutes* and *Bacteroidetes* (29.2% ± 25.2% and 13.1% ± 14.5% versus 12.5% ± 19.2% and 1.3% ± 6.2%) (Fig. 7 and Fig. S7).

We further compared the gut bacterial abundance at different taxonomic levels between specialists and generalists by linear discriminant analysis effect size (LEfSe) analysis (Fig. S8). At the class level, three specialists harbored more *Bacteroidia* and *Gammaproteobacteria* than did generalists. At the order level, specialists showed more *Bacteroidales* and unclassified *Gammaproteobacteria*, whereas generalists were dominated by *Rickettsiales*. At the family level, specialists possessed more *Porphyromonadaceae* and unclassified *Gammaproteobacteria*, while generalists had more *Rickettsiaceae*. At the genus level, specialists harbored a higher abundance of *Dysgonomonas*, *Enterococcus*, and unclassified *Gammaproteobacteria*, whereas generalists had more *Acetobacter*, *Gluconobacter*, and *Wolbachia*. This result suggested that bacterial communities in different categories are compositionally differentiated between the specialist and generalist drosophilid flies.

### Functional differentiation of bacterial communities between specialists and generalists.

Functional profiles of the gut bacterial communities were further predicted by Phylogenetic Investigation of Communities by Reconstruction of Unobserved States (PICRUSt) with the Kyoto Encyclopedia of Genes and Genomes (KEGG) database. The prediction accuracy estimated by the Nearest Sequenced Taxon Index (NSTI) was 0.051 ± 0.001, indicating a high PICRUSt prediction for most communities. In total, 5,653 KEGG orthologs (KOs) were predicted to be involved in the bacterial communities and further mapped to seven first-level, 41 second-level, and 276 third-level functional groups of KEGG pathways. Among these KOs and functional groups, 573 (10.1%; enriched in specialists versus generalists, 104 versus 469) KOs and 7 (100%; 4 versus 3) first-level, 26 (63.4%; 16 versus 10) second-level, and 67 (24.3%; 36 versus 31) third-level functional groups were significantly differentiated between the specialist and generalist drosophilid flies (LEfSe analysis, *P < *0.05; linear discriminant analysis [LDA] score > 2.5 for the KOs, LDA score > 3 for the KEGG pathways) (Tables S3 and S4). Notably, amino acid and energy metabolic pathways were the most diverse functional groups. Seven amino acid-related pathways are involved in the metabolism of 11 amino acids, including eight essential (arginine, histidine, isoleucine, leucine, phenylalanine, threonine, tryptophan, and valine) and three nonessential (glycine, proline, and serine) amino acids that were enriched in at least two specialists flies, whereas only two pathways involved in the metabolism of one amino acid (lysine) were enriched in generalists. In contrast, two energy metabolic pathways involved in methane and nitrogen metabolism were enriched in specialist flies, whereas five pathways involved in carbon fixation, photosynthesis, oxidative phosphorylation, and sulfur metabolism were enriched in generalist flies.

## DISCUSSION

Our results showed that the gut bacterial community compositions in *Colocasiomyia* were mainly affected by combined environmental factors indicated by the year. In previous studies, the host phylogeny and diet were identified as the main factors shaping bacterial communities in many animals (31). These two factors contributed to a small fraction of the community diversity may be due to the similar genetic backgrounds in *Colocasiomyia* flies, the movements of flies between host plants, and the similar nutrient contents of the host plants. In the meta-analysis using more species from different lineages, the bacterial communities from flies with different dietary preferences were clustered by dietary utilization but not species phylogeny. The phylogenetic effect was also scarcely detected in other *Drosophila* studies (32, 33), despite being observed frequently in many animals (34). The gut bacterial communities were affected both by bacteria acquired from the diet and by the dietary content. The compositional and functional comparison of gut communities between specialists and generalists suggested that dietary content differences in the proteins, carbohydrates, vitamins, and plant secondary metabolites all can shape the fly bacterial communities.

Data from flies with different dietary specializations showed that the specialist species tended to have more novel bacterial species than did the generalist flies. A previous study showed more than 90% of termite bacterial OTUs were novel (35). Some of these novel bacteria have been characterized as playing a pivotal role in cellulose degradation (36). Thus, we speculate that the high proportion of novel bacteria might play a role in the dietary adaptation of specialists. In this study, one of the predominant novel bacteria in Araceae-feeding and cactophilic flies belongs to *Ruminococcaceae*. Many members of the *Ruminococcaceae* present functions involved in polysaccharide and cellulose degradation (37–39) and are strongly associated with herbivores and omnivores (40). These findings suggest that complex carbohydrate utilization in the dietary specialization of specialist drosophilid flies was partly due to novel bacteria.

Compositional and functional differentiation of bacterial communities between the specialist and generalist flies were also significant in this study. The gut bacterial communities in drosophilids consisted of three major phyla, namely, *Proteobacteria*, *Firmicutes*, and *Bacteroidetes*, as reported for other insects (*Proteobacteria*, 62.1%; *Firmicutes*, 20.7%; and *Bacteroidetes*, 6.4%) (41). The specialist flies harbored higher proportions of *Firmicutes* and *Bacteroidetes*, whereas the generalist flies possessed higher proportions of *Proteobacteria*. Similarly, functional differentiation, especially the differentiation of metabolic pathways, has also been observed between the specialists and generalists. Dietary utilization between specialists and generalists, as discussed below, might lead to the compositional and functional differentiation of the gut bacterial communities in flies.

First, carbohydrate contents in the natural diets of fly species in this study were different. The generalists utilized rotten fruit rich in sugars, while the specialists exploited diets with more structurally complex carbohydrates, such as pectin in the pollen of Araceae plants, chitin in mushrooms, and glucans in cacti (28, 42–45). In contrast to sugars, which can be directly absorbed or digested by animals, structural complex carbohydrates can be utilized by animals only after being broken into smaller subunits by microorganisms (46–49). Our data showed that the generalist drosophilid flies harbored abundant *Proteobacteria*, especially *Acetobacter* and *Gluconobacter*, which are mainly involved in sugar metabolism (50). Enrichment of *Proteobacteria* has also been found in laboratory-reared *Drosophila* and mice fed sugar-rich diets (22, 27, 43, 51, 52). In contrast to generalist flies, specialist flies harbored more *Bacteroidetes* and *Firmicutes*, such as *Bacteroides*, *Ruminococcus*, and *Prevotella*, which possess the enzyme repertoire for degrading complex carbohydrates (53–57). These findings support the idea that the gut bacterial community plays an evident role in the dietary carbohydrate utilization of flies. To determine whether vertebrates have similar influences of carbohydrate utilization on the gut bacterial community, we examined the bacterial community divergence among herbivores, carnivores, and omnivores based on the data collected by Youngblut et al. (58). The results showed that carnivores and omnivores harbored abundant *Proteobacteria*, whereas herbivores tended to harbor more *Bacteroidetes* and *Firmicutes* (Fig. S9). The higher abundance of *Proteobacteria* in carnivores and omnivores might reflect the fact that the primary carbohydrate sources in these vertebrates are digestible carbohydrates, such as starch and glycogen, which can be easily broken down into sugars (59, 60) and subsequently utilized by *Proteobacteria* (51, 61). In contrast, the microbiotas rich in *Bacteroidetes* and *Firmicutes* in herbivores might indicate that the primary carbohydrate sources in these animals are indigestible, such as cellulose and chitin (62, 63). Together, the enrichment of gut bacteria is associated with dietary carbohydrate utilization in animals.

Second, the diets of specialists contain a higher portion of proteins than those of generalists (21, 43, 64–66). Previous studies have shown that *Bacteroidetes* possess the highest proteolytic activity among phyla (67, 68). In this study, *Bacteroidetes* were indeed enriched in the bacterial communities of specialists. Additionally, functional prediction of bacterial communities showed that KEGG pathways of amino acid and nitrogen metabolism were enriched in the bacterial community of specialists. Altogether, these results provide evidence that specialist drosophilid flies may utilize more proteolytic bacteria than generalists to adapt to diets with a higher ratio of proteins.

Third, the demand for vitamin B_12_ from gut bacteria is higher in specialist flies than in generalist flies. Vitamin B_12_ is the sole vitamin that can be synthesized by a limited number of bacteria (69) and is absent in most plant-derived foods (70). The source of vitamin B_12_ for herbivorous animals is mainly bacterium-fermented foods and/or the gut bacterial community (70). In contrast to generalist flies, which utilize a wide range of dietary substrates, including fermented fruits and vegetables, specialists consume limited dietary substrates. In this regard, the demand for vitamin B_12_ obtained from the gut bacterial community in specialists would be higher than that in generalists. As expected, we found that two genera, *Dysgonomonas* and *Enterococcus*, with biosynthetic activities of vitamin B_12_ (71, 72) were enriched in the bacterial communities of the specialist drosophilid flies.

Finally, the demand for the detoxification of secondary metabolites is higher in specialists than in generalists. Among the bacteria enriched in specialists, *Enterococcus* has been documented to be associated with secondary metabolite detoxification or resistance in other studies (5). For example, a latex-tolerant *Enterococcus* species could form biofilms in the guts of Hyles euphorbiae and Brithys crini moths to resist toxic latexes and alkaloids of host plants (73). Considering that Araceae plants contain latexes (74) and cacti contain alkaloids (75), it is possible that the enriched *Enterococcus* is also involved in toxin resistance in the specialist drosophilid flies. Additionally, our functional enrichment analyses showed that the KEGG pathways related to the metabolism of secondary metabolites were enriched in the bacterial communities of specialist species. These results suggest that the gut bacterial communities of specialist flies are involved in the detoxification of secondary metabolites.

In conclusion, the comprehensive comparison of closely related specialist and generalist drosophilid flies demonstrates that the compositional and functional differentiation of gut bacterial communities is associated with the different dietary utilization and demands of fly hosts. This study offers a framework for how gut bacteria may contribute to the dietary specialization of drosophilid flies, at least in the genera *Colocasiomyia* and *Drosophila*, studied here.

## MATERIALS AND METHODS

### *Colocasiomyia* fly collection and sample preparation.

Adult *C. alocasiae* and *C. xenalocasiae* flies were collected directly from the flowers of the two host plants, *A. odora* and *C. formosana* (Fig. 1a and b), with an aspirator in May from 2013 to 2016 in Wulai, New Taipei City, Taiwan (24°81′N, 121°51′E). Flies collected from individual host plants were transferred into the sterile glass vials with 1% agar medium. At the same time, the surface of the host plants where the flies were sampled was swabbed using cotton swabs. Then, the swabs were inserted into a 1.5-mL Eppendorf tube with 1 mL of sterile water. The two *Colocasiomyia* species were identified by examining the difference in costal bristle patterns (76) (Fig. S10a and b) under a microscope. The external bacteria of flies were removed by the modified method of Chandler et al. (22). In brief, the flies were washed in a 1.5-mL Eppendorf tube containing 1 mL of 70% ethanol and 0.1-mm glass beads by vortexing for 20 s and rinsed with water three times with a similar procedure. The guts dissected from five males or five females of each species collected from a single host plant were pooled for further DNA extraction. Flies collection, host plant swabs, and fly gut dissection were completed within 6 h. In total, 16 and 12 bacterial communities were sampled from *C. alocasiae* and *C. xenalocasiae*, respectively (Table S1).

### DNA extraction and 16S rRNA library preparation.

Bacterial DNA was extracted from 50-μL samples using the Gentra Puregene Yeast/Bact. kit (Qiagen, Hilden, Germany) according to the manufacturer’s instructions. Illumina (San Diego, CA, USA) MiSeq paired-end sequencing was performed to obtain the 16S rRNA sequence. For library preparation, the hypervariable V3-V4 region of the 16S rRNA gene was amplified by two-step PCR amplification using the modified dual-index sequencing strategy (77). The first round of PCR amplification was carried out using the KAPA HiFi HotStart ReadyMix kit (Roche, Basel, Switzerland) and a pair of overhang adapter primers (5′-TCGTCGGCAGCGTCAGATGTGTATAAGAGACAGCCTACGGGNGGCWGCAG-3′ and 5′-GTCTCGTGGGCTCGGAGATGTGTATAAGAGACAGGACTACHVGGGTATCTAATCC-3′). The 25-μL PCR mix contained a final concentration of 1× KAPA HiFi HotStart ReadyMix, a 0.2 μM concentration of each primer, and 0.5 ng/μL extracted bacterial DNA. The thermocycling profile included a 95°C initial hold for 3 min followed by 25 cycles of denaturation at 95°C for 30 s, annealing at 55°C for 30 s, and extension at 72°C for 30 s and a final 72°C extension for 4 min. Twenty-five microliters of V3-V4 amplicons was purified using AMPure XP beads (Beckman Coulter, Brea, CA, USA) to remove excess primers and primer dimers. Secondary index PCR was performed using the Nextera XT Index kit (Illumina). Twenty-five microliters of PCR contained 1× KAPA HiFi HotStart ReadyMix, a 0.5 μM concentration of each Nextera XT index primer, and 2.5 μL of AMPure XP bead-purified DNA. The thermocycling profile included a 95°C initial hold for 3 min followed by 8 cycles of denaturation at 95°C for 30 s, annealing at 55°C for 30 s, and extension at 72°C for 30 s and a final 72°C extension for 5 min. All PCRs were monitored for laboratory contamination using sterile water as a negative control. Ten-nanogram portions of PCR products from each sample were pooled, and then 2 × 300-bp paired-end sequencing was performed using the Illumina MiSeq system.

### Sequence data processing.

Paired-end reads of bacterial communities from *Colocasiomyia* flies and their host plants were processed using mothur 1.42 (78) according to MiSeq standard operating procedures (SOP) (Fig. S1). Raw reads were filtered and aligned into contigs with default parameters. Contigs longer than 500 bp or with more than three ambiguous bases were excluded. The remaining contigs were further assigned to unique sequences to represent multiple identical reads. The unique sequences were aligned against the SILVA reference database (update in 2017) (79). The poorly matched sequences and the nonaligned bases were further removed. The remaining unique sequences within a 1% difference were then preclustered. Chimeric sequences were identified using VSEARCH (2.11.1) (80) and discarded. Subsequently, the selected sequences were clustered into OTUs at a similarity threshold of 97% after being classified as bacterial 16S rRNA by the Bayesian classifier (81) against the Greengenes database (version gg_13_8_99) (82) with a bootstrap confidence threshold of 80%. Finally, to ensure that the OTUs were supported by sufficient reads and to consider different read depths in the subsequent meta-analysis, the OTUs with relative abundances greater than 0.02% (approximately 100 reads in the *Colocasiomyia* data) were used for further analyses (Table S1).

### Bacterial community analysis in *Colocasiomyia* and host plants.

To test if the bacterial communities of flies and host plants were associated, the correlation of the bacterial OTU abundance between paired samples of flies and host plants was assessed by Spearman’s rank correlation analysis. The biodiversity of bacterial communities from *Colocasiomyia* and host plants was measured by alpha-diversity metrics, including the number of OTUs and Shannon’s index (83), and by beta-diversity metrics, including weighted UniFrac dissimilarity (84), unweighted UniFrac dissimilarity (84), Bray-Curtis dissimilarity (85), Jaccard distance (86), and Theta YC distance (87), using mothur with the default settings. To visualize the bacterial community differences, multidimensional scaling, NMDS (88, 89), and PCoA (90) were applied. NMDS based on a Bray-Curtis dissimilarity matrix was performed using the vegan package of R with 999 iterations. PCoA based on weighted and unweighted UniFrac dissimilarity were performed using the default setting in mothur. The relative contributions of various factors, including year, fly species, fly sex, and host plant, to the variation in bacterial communities of *Colocasiomyia* were evaluated by permutational multivariate analysis of variance (PERMANOVA; Adonis) with 999 permutations using the vegan package (91) in R (version 3.6.1) (92, 93) based on the beta-diversity measures. All figures were plotted in R.

### Meta-analysis of the gut bacterial community in *Drosophila*.

The data sets applied in the meta-analysis in this study were selected to meet the following four criteria: (i) 16S rRNA gene sequences were obtained by next-generation sequencing (NGS) methods, including Illumina MiSeq and Roche-454; (ii) samples were from natural populations; (iii) for statistical power, only bacterial communities for each species with a sample size larger than five were included; and (iv) the dietary substrates of fly species were clearly described in published papers. The gut bacterial 16S rRNA sequences of natural populations of eight *Drosophila* species, including four mushroom-feeding species (*D. falleni *[23, 29], *D. neotestacea* [23, 29], *D. putrida* [23, 29], and *D. recens* [23]), one cactus-feeding species (*D. nigrospiracula* [28]), and three generalist species (D. melanogaster [24, 27], *D. simulans* [27], and *D. suzukii* [25, 26]), were obtained from the NCBI Sequencing Read Archive (SRA) and Metagenomic Rapid Annotations using Subsystems Technology (MG-RAST) (94) (Table S5). Raw sequencing data from each study were processed separately to obtain contigs using the general sequence processing commands in mothur according to the MiSeq or 454 SOPs with default parameters as described on the mothur website (https://mothur.org/). The data were processed using a similar procedure performed for the *Colocasiomyia* data to generate tables of OTU counts and taxonomic assignments. The alpha diversity of each bacterial community was measured by Shannon’s index using the OTU count table. Beta diversity was measured by Bray-Curtis dissimilarity and rescaled by NMDS using the genus count table. To build the dendrogram of bacterial communities of fly species, OTU counts were first combined by species. Then, the similarities of combined bacterial communities were estimated by calculating Bray-Curtis dissimilarity and clustered by UPGMA (95). The phylogenetic tree of drosophilid species was based on the phylogenies reconstructed in previous studies (96, 97).

Statistical comparisons of bacterial communities among the drosophilid species were performed by the Kruskal-Wallis test followed by Dunn’s test with the Bonferroni correction. Differences in the main gut bacterial phyla between specialist and generalist flies were determined with the Mann-Whitney *U* test. All statistical analyses were performed in R. The compositional differentiation of bacterial communities between specialists and generalists was defined by an LDA effect size (LEfSe) (98) score larger than 3.0.

### Functional prediction based on bacterial community compositions.

The functional profiles of bacterial communities were predicted using PICRUSt (99). The accuracy of PICRUSt prediction was estimated using the NSTI. The lower the NSTI score, the more accurate PICRUSt’s predictions are. A score less than 0.06 indicates a reliable prediction that a high number of reads can be assigned to reference sequences. The relative abundances of bacterial genera were transformed into counts of KOs and then summarized at KEGG pathway levels 1, 2, and 3. Differential molecular functions and KEGG pathways between the fly species with different diets were defined by LEfSe scores (LDA scores of >2.5 and >3.0, respectively).

### Data availability.

Raw sequencing reads were deposited in the NCBI SRA under BioProject ID PRJNA699707. All the data sets used for the meta-analysis in this study are presented in Table S5 in supplemental file 2.
