# Supplementary material for: Dietary Utilization Drives the Differentiation of Gut Bacterial Communities between Specialist and Generalist Drosophilid Flies
Source: Microbiol Spectr. 2022 Jul 11;10(4):e01418-22. doi: 10.1128/spectrum.01418-22 (PMC9431182; doi:10.1128/spectrum.01418-22)
Supplement: Supplemental file 1 — Fig. S1 to S10. Download spectrum.01418-22-s0001.pdf, PDF file, 6.1 MB [file spectrum.01418-22-s0001.pdf]

Supplementary Material of

Dietary utilization drives the differentiation of gut bacterial communities between specialist and generalist flies

Jia-Syuan Chen[1], Shun-Chern Tsaur[2], Chau-Ti Ting[1,3,4,5,6], and Shu Fang[7]

1. Department of Life Science, National Taiwan University, Taipei 10617, Taiwan, ROC
2. Center for General Education, National Taiwan University, Taipei 10617, Taiwan, ROC
3. Institute of Ecology and Evolutionary Biology, National Taiwan University, Taipei 10617, Taiwan, ROC
4. Center for Biotechnology, National Taiwan University, Taipei 10617, Taiwan, ROC
5. Center for Developmental Biology and Regenerative Medicine, National Taiwan University, Taipei 10617, Taiwan, ROC
6. Genome and Systems Biology Degree Program, National Taiwan University and Academia Sinica, Taipei 10617, Taiwan, ROC
7. Biodiversity Research Center, Academia Sinica, Taipei 11529, Taiwan, ROC

Co-corresponding authors

Chau-Ti Ting +886 2 33662522; ctting@ntu.edu.tw

Shu Fang +886 2 27899562; zofang@sinica.edu.tw

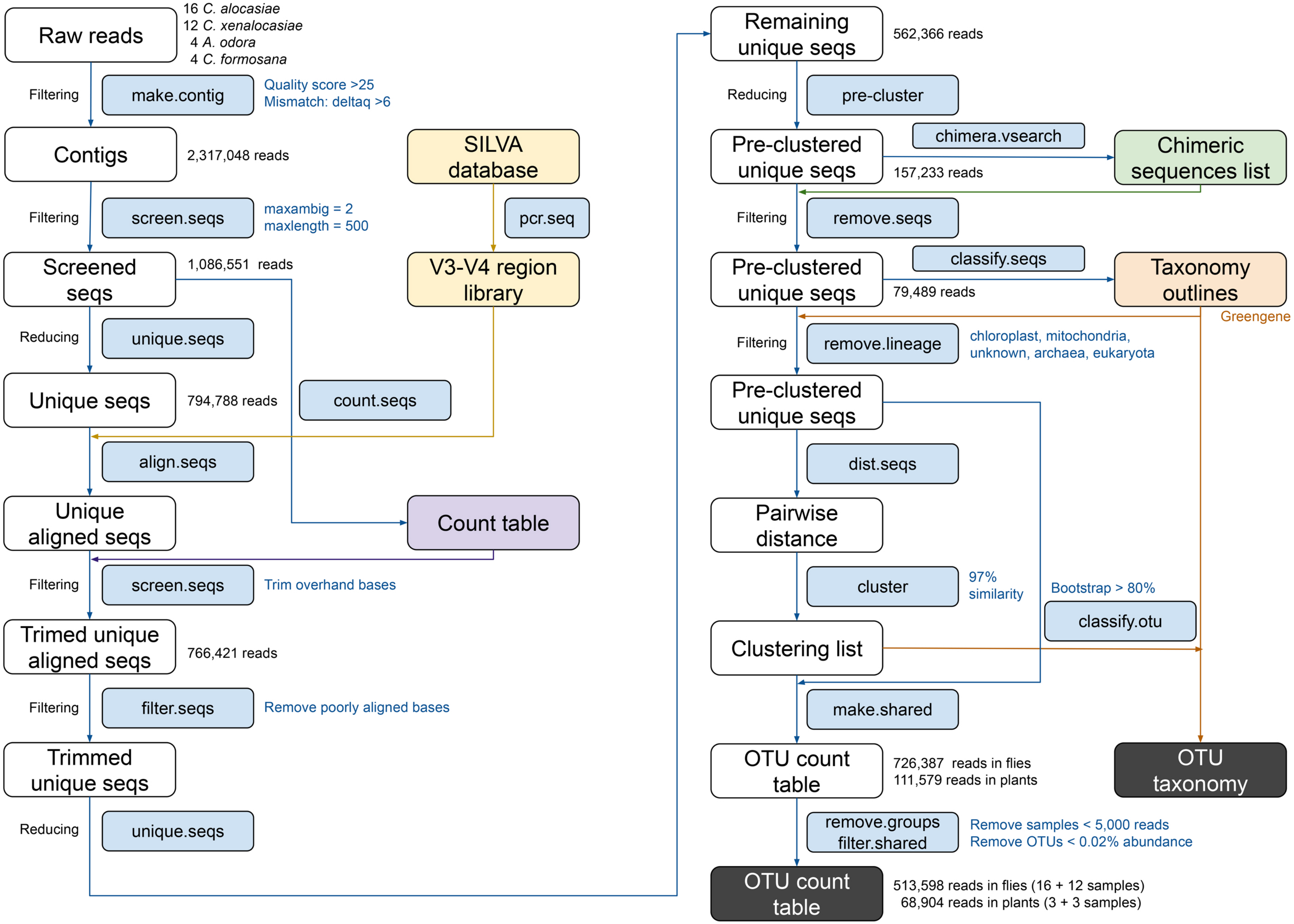

Fig. S1 Data processing and analysis pipelines.

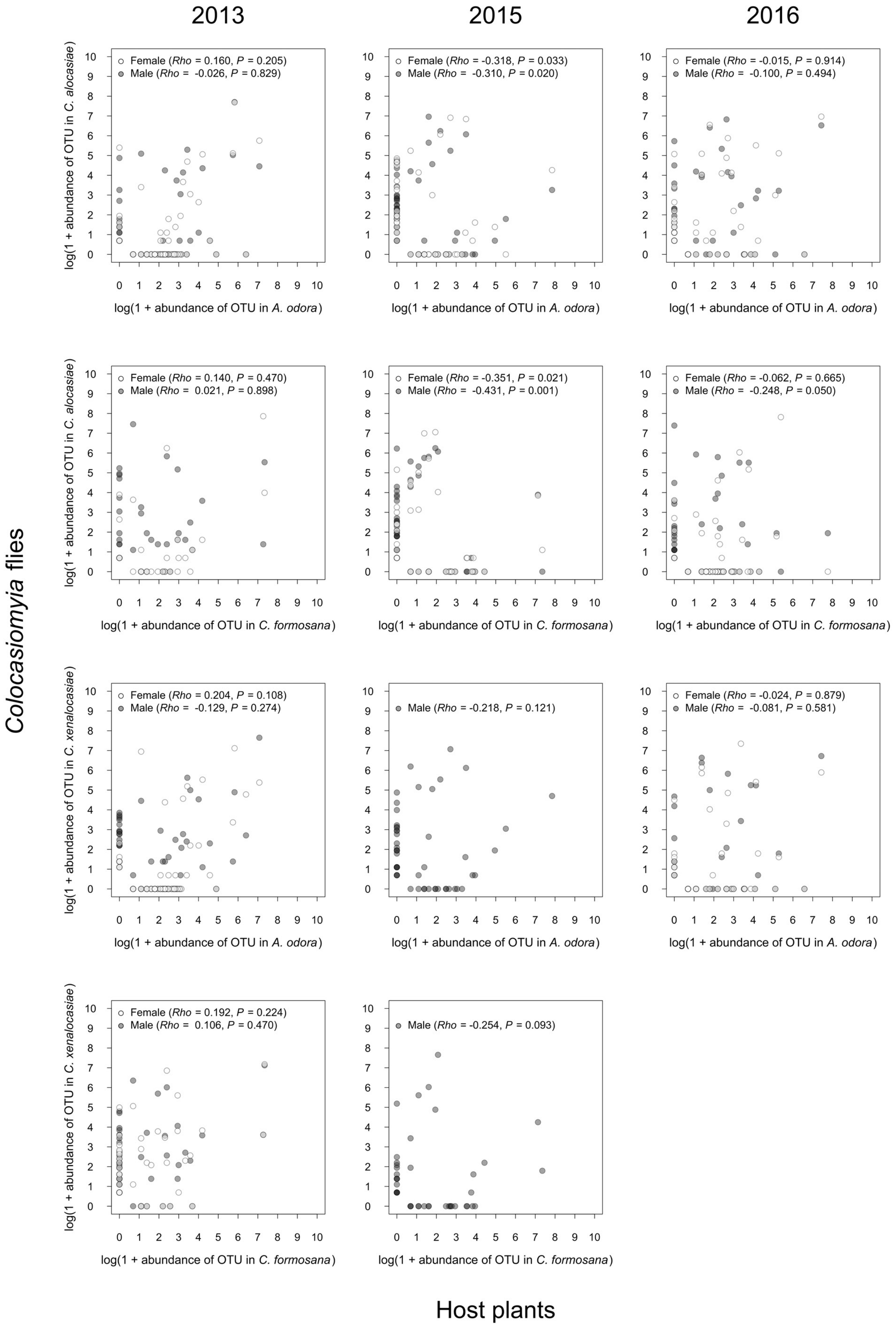

Fig. S2 Pairwise comparison of the abundance of bacterial operational taxonomic units (OTUs) between *Colocasiomyia* flies and host plants. The correlation between flies and host plants was estimated by Spearman's rank correlation coefficients.

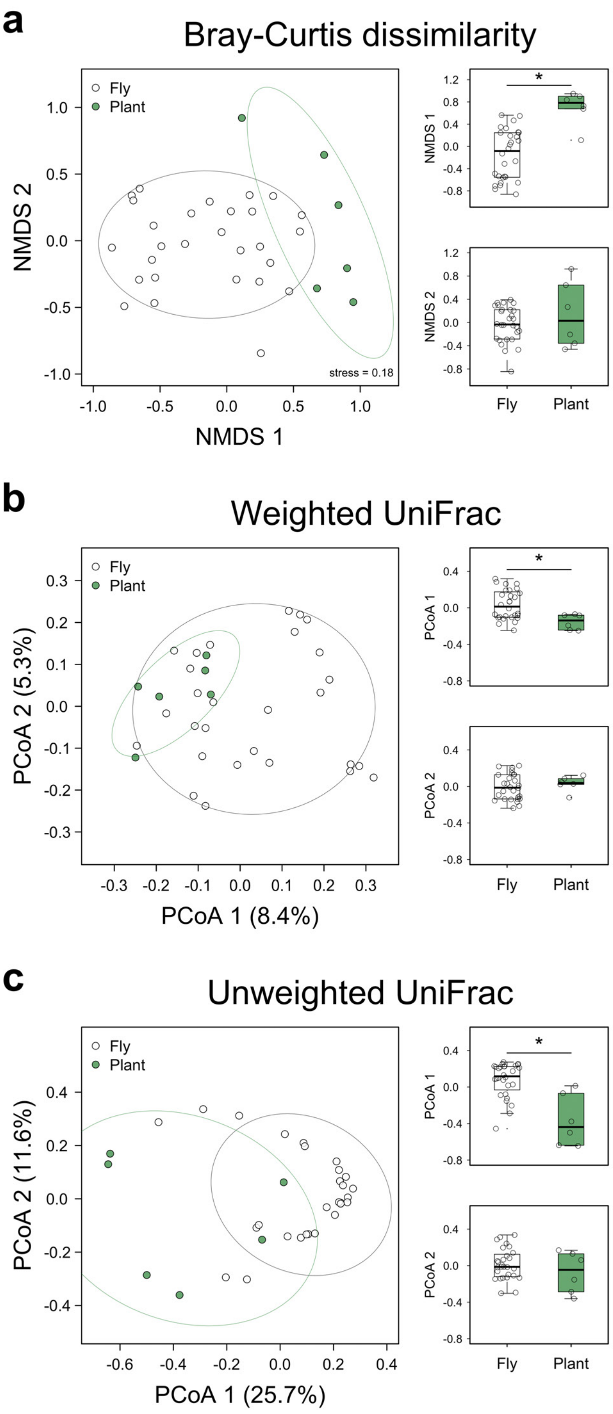

Fig. S3 Multidimensional scaling of the bacterial communities in *Colocasiomyia* flies and host plants. (a) Non-metric multidimensional scaling (NMDS) was based on Bray-Curtis dissimilarity. (b and c) Principal coordinate analyses (PCoA) were based on Unweighted and Weighted UniFrac dissimilarity. The difference between fly and host plant samples was tested by the Mann-Whitney U test. The asterisk indicates statistical significance ($P < 0.05$).

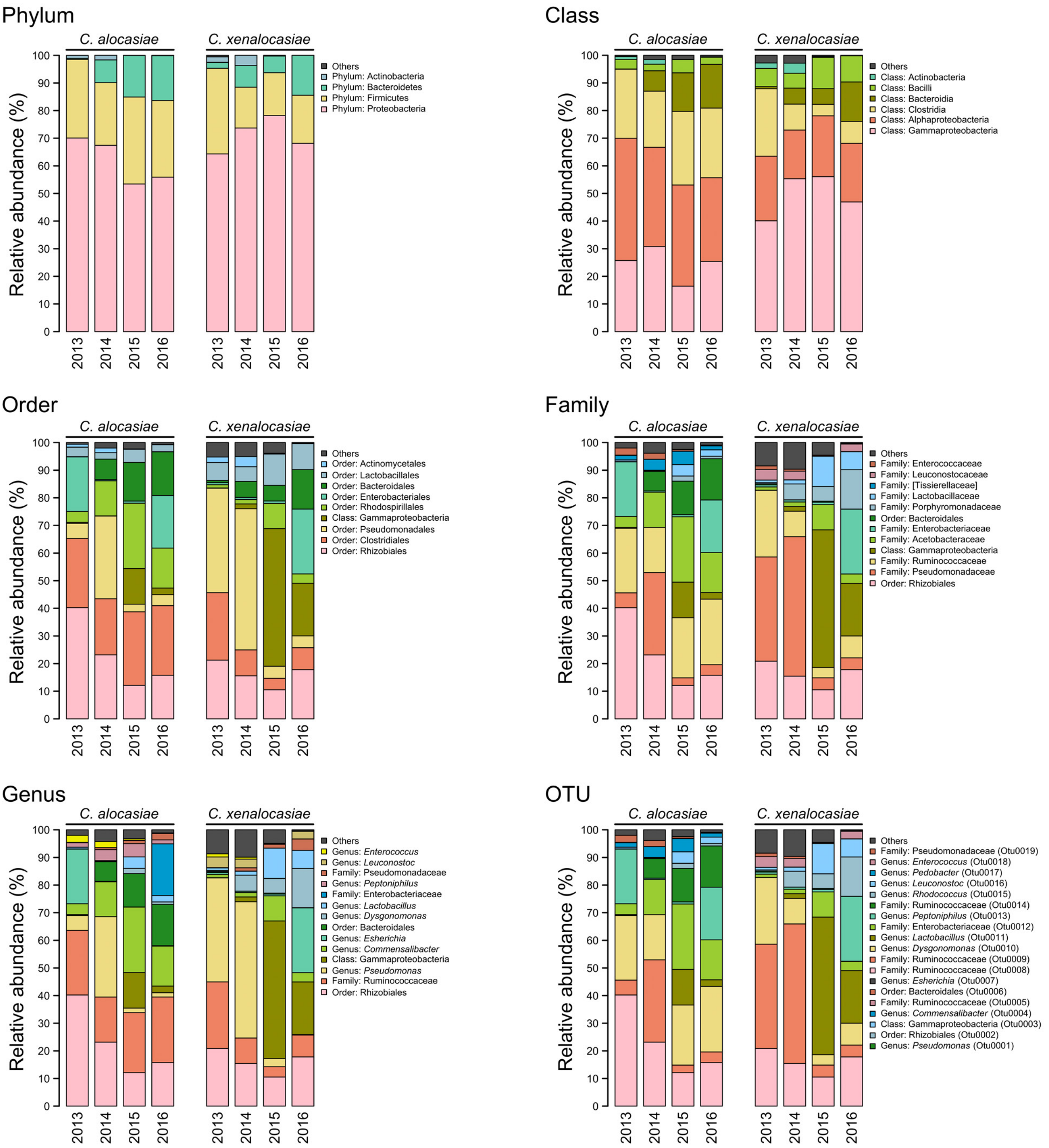

Fig. S4 Relative abundances of different bacterial categories identified in *Colocasiomyia* fly guts across years.

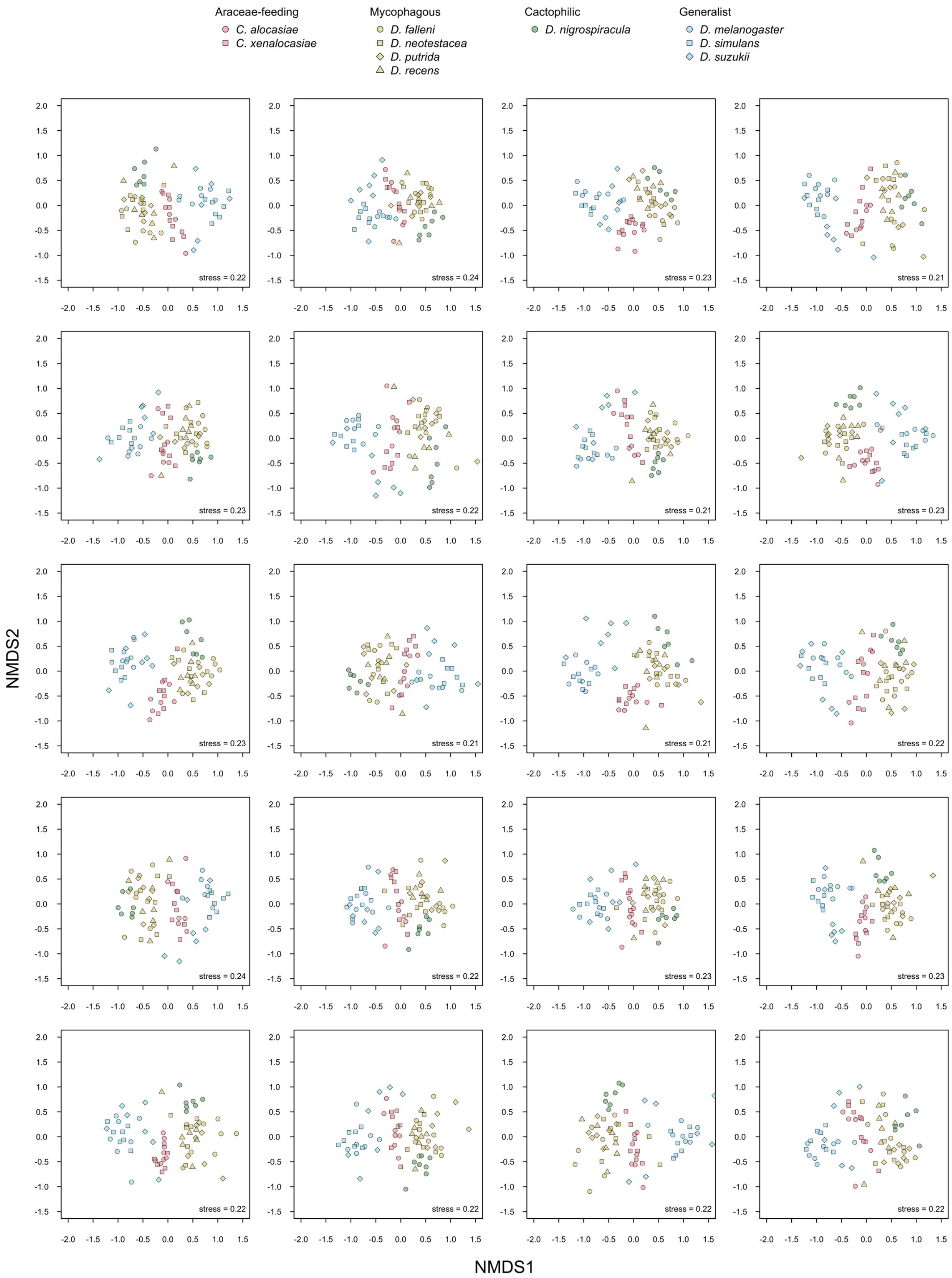

Fig. S5 Nonmetric multidimensional scaling (NMDS) plot of randomly resampled gut bacterial communities from different drosophilid species (sample size = 7).

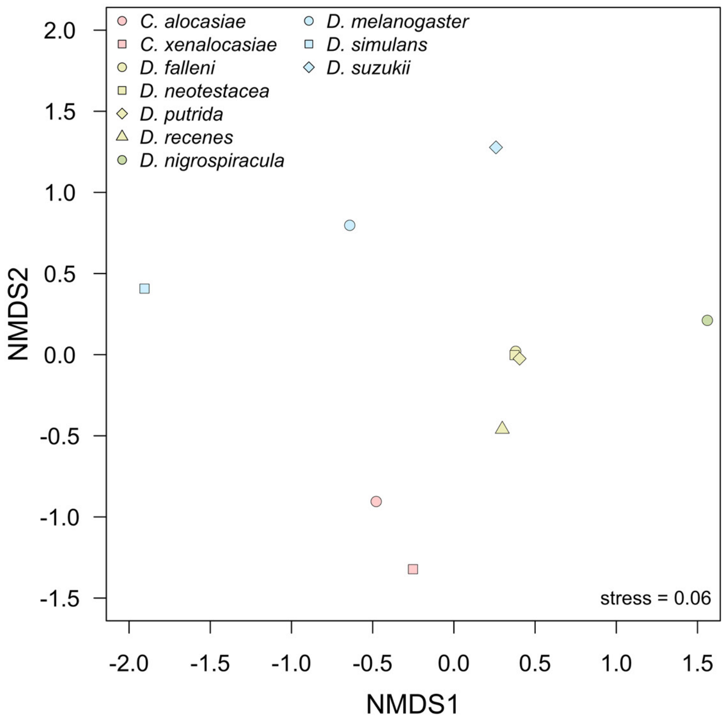

Fig. S6 Nonmetric multidimensional scaling (NMDS) plot of average gut bacterial communities in different drosophilid species.

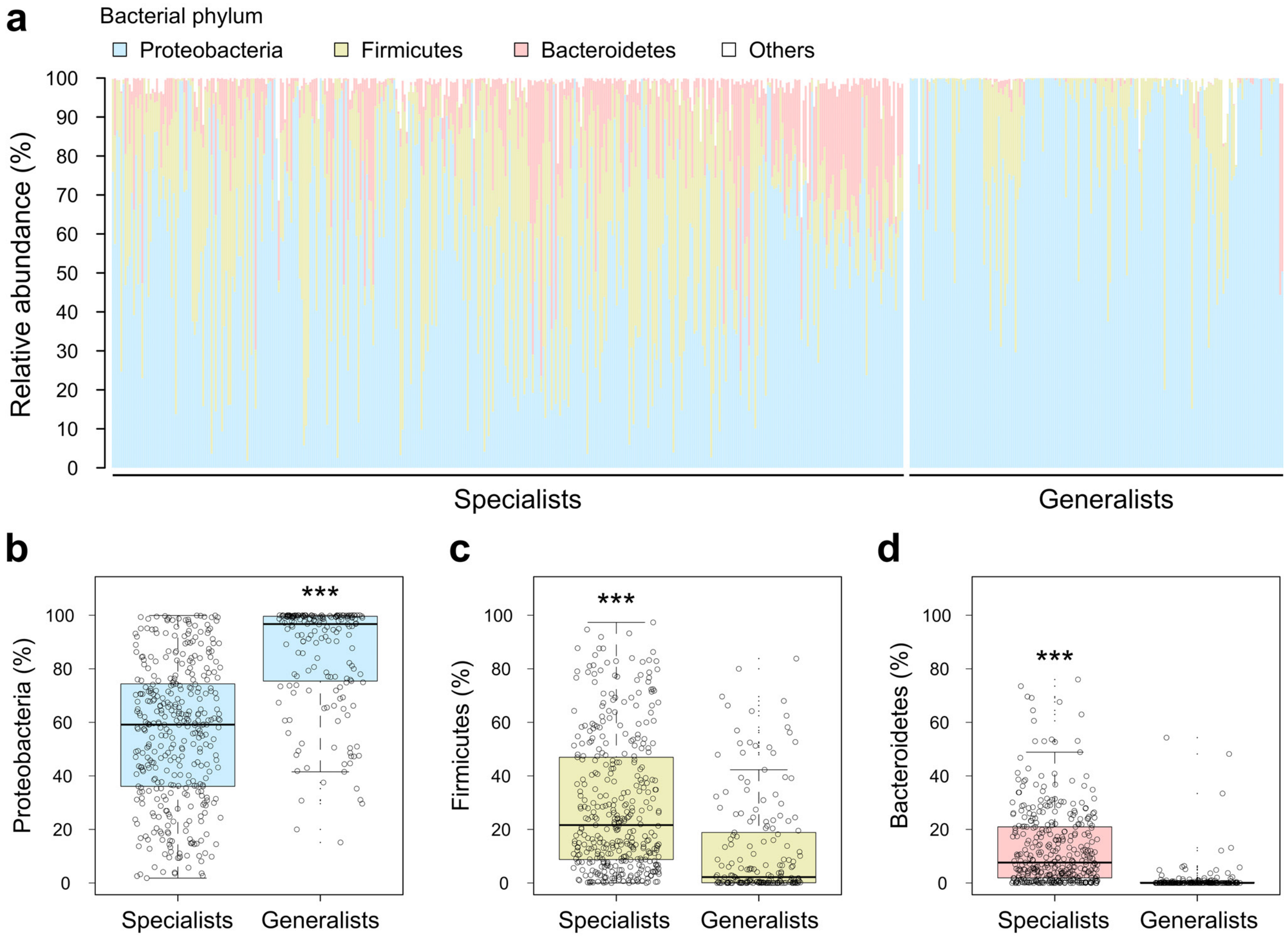

Fig. S7 Relative abundances of gut bacterial phyla in specialist and generalist flies. (a) All bacterial phyla in each community. (b) Proteobacteria. (c) Firmicutes. (d) Bacteroidetes. Asterisks above the boxes indicate significant differences (* $P < 0.01$; ** $P < 0.001$; *** $P < 0.0001$) determined by the Mann-Whitney *U* test.

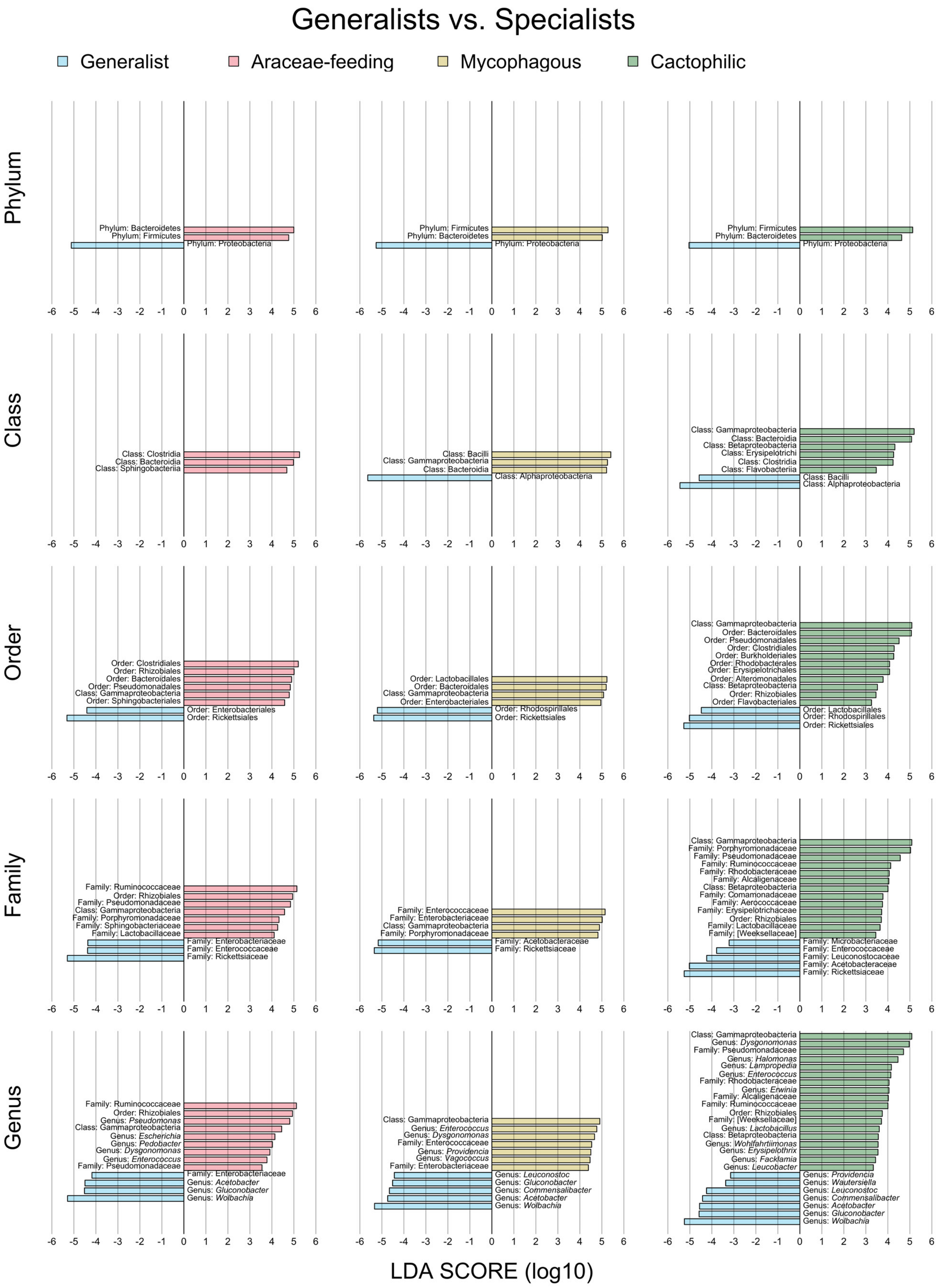

Fig. S8 Enriched bacteria in specialist and generalist flies revealed by the LEfSe approach. Histograms show linear discriminant analysis (LDA) values of enriched bacteria at different taxonomic levels.

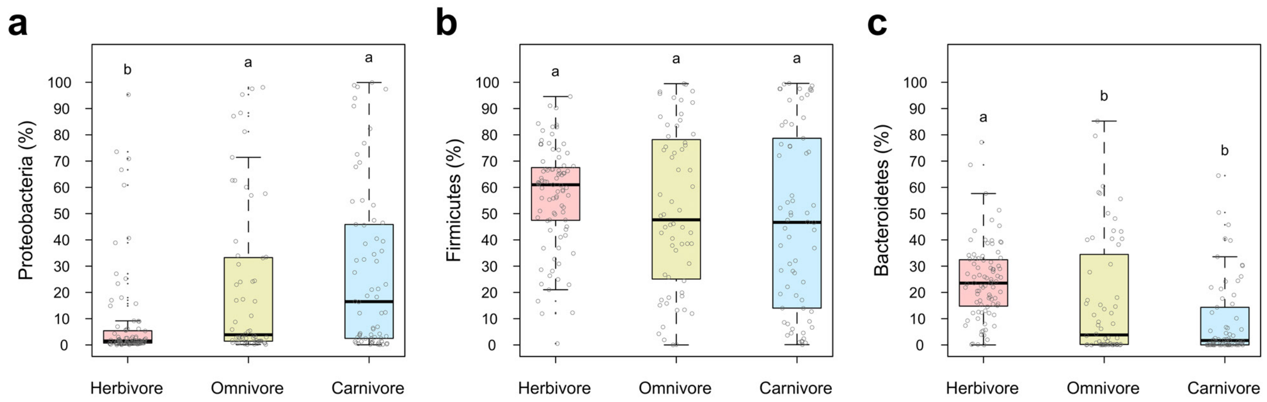

Fig. S9 Relative abundances of the main gut bacterial phyla in herbivores, omnivores, and carnivores. (a) Proteobacteria. (b) Firmicutes. (c) Bacteroidetes. The dataset is from Youngblut et al. (2019, Nat. Commun. 10:2200). Different letters above the boxes indicate significant differences at $P < 0.05$ determined by the Kruskal-Wallis test followed by Dunn's test with Bonferroni correction.

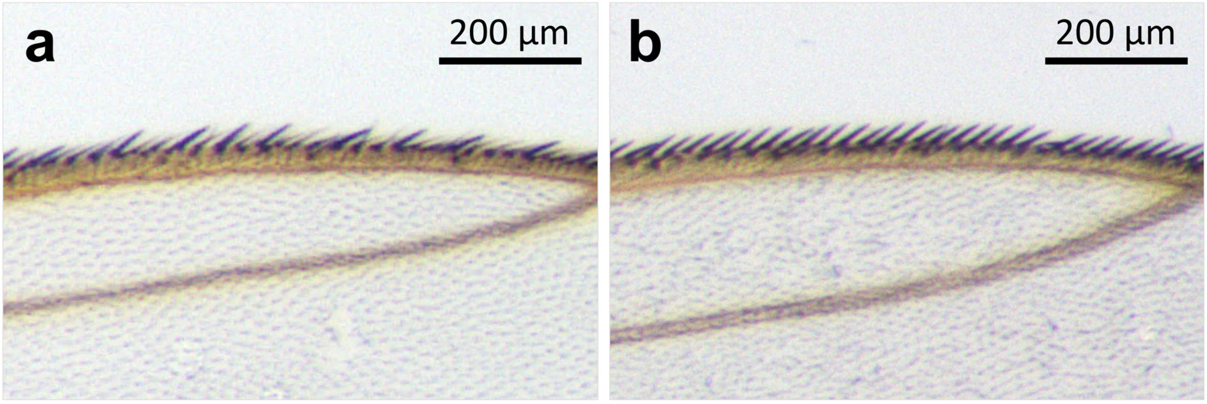

Fig. S10 Bristle patterns along the anterior wing margin (costa vein) of (a) *C. alocasiae* and (b) *C. xenalocasiae*.
